# Combined Posterior and Anterior Ankle Arthroscopy

**DOI:** 10.1155/2012/693124

**Published:** 2012-10-08

**Authors:** Peter E. Scholten, C. Niek van Dijk

**Affiliations:** ^1^Department of Orthopaedic Surgery, Kliniek Klein Rosendael, Rosendaalselaan 30, 6891 DG Rozendaal, The Netherlands; ^2^Department of Orthopaedic Surgery, Academic Medical Center, University of Amsterdam, 1105 AZ Amsterdam, The Netherlands

## Abstract

Treatment of combined anterior and posterior ankle pathology usually consists of either combined anterior and posterior arthrotomies or anterior ankle arthroscopy with an additional posterolateral portal. The first technique bears the risk of complications associated with the extensive exposure, the latter technique provides limited access to the posterior ankle joint. A case is described of combined anterior and posterior arthroscopy, with the patient lying prone and then turned supine, addressing both anterior and posterior ankle pathologies in one tempo. This minimally invasive combined approach allows quick recovery and early return to work and sports activities.

## 1. Introduction

In the early days of the orthopaedic era the ankle joint was regarded unsuitable for arthroscopy because of its typical anatomy [1]. Nowadays it is an established treatment of choice for most ankle pathologies. Routinely, anteromedial and anterolateral portals are used. An additional posterolateral portal has been advocated in cases of posterior ankle pathology, but it has usually been viewed as an accessory rather than the main working portal [2]. Arthroscopic procedures which are performed using this portal are not easy [3]. The use of a 2-portal endoscopic approach to the posterior ankle with the patient in the prone position gives excellent access for the examination and treatment of posterior ankle pathology [4].

 When both posterior and anterior ankle pathology coexist, the surgeon may consider an open anterior and posterior arthrotomy. An open approach by both anterior and posterior incisions has an increased risk of complications and a prolonged rehabilitation period. An anterior arthroscopic approach with an accessory posterior portal, will give good access to the anterior joint, but compromises access to the posterior joint.

This paper describes a patient with combined anterior and posterior ankle pathology, admitted to the coauthors hospital. A combined posterior and anterior arthroscopic approach was used, initially with the patient prone for the posterior arthroscopic procedure and then turned supine for the anterior arthroscopic procedure, both in the same surgical session. This combined arthroscopic approach gives excellent access to both the anterior and posterior aspects of the ankle. 

## 2. Patient and Methods

A 21-year-old professional soccer player, who was operated for a medial malleolar fracture of his right ankle 10 years before, developed pain and swelling of the medial side of his right ankle. He was diagnosed as having a flexor hallucis longus (FHL) tendinitis and a reactive posturethritis arthritis of the ankle. He was treated with local application of ice and oral nonsteroidal anti-inflammatory drugs. Five months later, he returned to his sporting activities but suffered an injury to his ankle after being tackled by an opponent during soccer. The ankle was forced in plantar flexion. A relapse of the FHL tendinitis and the reactive arthritis was seen and eventually appeared to be resistant to conservative treatment. Three months later he was referred to our outpatient department complaining of deep pain in the ankle in addition to localized anteromedial and posteromedial pain. 

 At physical examination, there were signs of diffuse synovitis of the right ankle. Dorsal and plantar flexion were only slightly reduced compared to the left ankle. In addition to tenderness over the medial malleolus, crepitus and recognizable pain were felt posterior of the medial malleolus over the flexor hallucis longus tendon. Forced plantar flexion produced recognizable pain posteriorly at the level of the talocrural joint. There was no evident ankle laxity. Muscle testing showed grade 5 muscle strength bilaterally. Neurologic and vascular examinations were normal.

**Figure 1:**
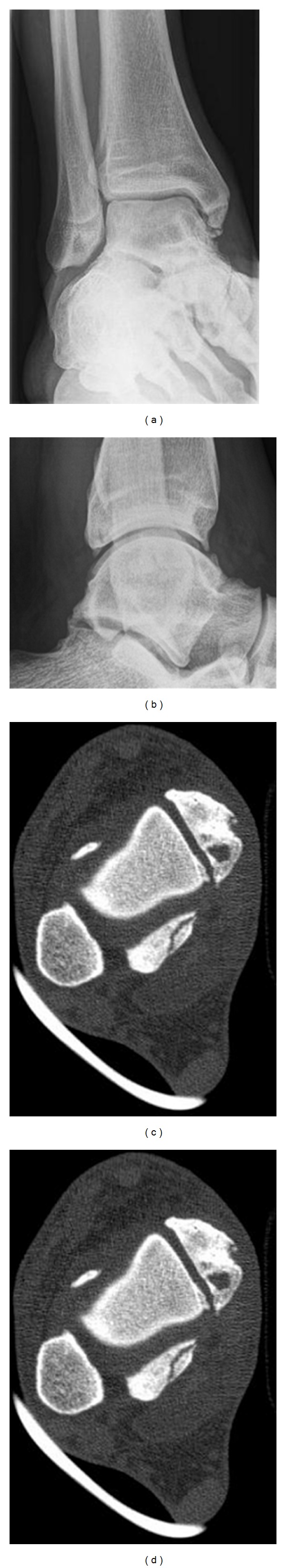
(a) AP radiograph showing medial malleolar ossicles. (b) Lateral radiograph showing anterior osteophytes. (c) and (d) Transversal CT slices showing the pseudarthrotic fragment of posterior distal tibial rim.

Radiographs (AP, lateral, and mortise X-ray views) and CT scan revealed several loose fragments and ossicles distal to the medial malleolus (Figures 1(a)-1(b)). The posterior tibial rim contained a pseudarthrotic fragment at the level of the flexor hallucis longus tendon (Figures 1(c)-1(d)).

The patient was diagnosed as having a nonunion of the posterior distal tibial rim with secondary FHL tendinitis causing posterior ankle pain. The anteromedial pain was explained by the presence of multiple ossicles distal and anterior to the medial malleolus with secondary synovitis. To address both the posterior as well as the anterior ankle compartment the patient was scheduled for a combined posterior and anterior ankle arthroscopy. It was planned to remove the nonunited fragment of the tibia and release the FHL tendon posteriorly, and to remove the loose ossicles and perform a synovectomy anteriorly.

**Figure 2:**
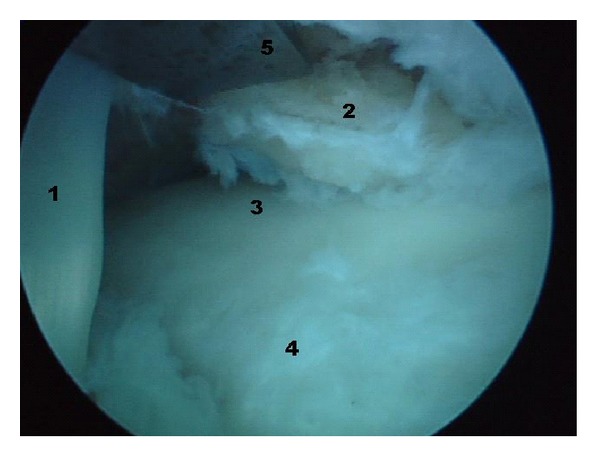
Endoscopic views posteromedial ankle joint. 1. Scarring of the flexor hallucis longus tendon. 2. Posteromedial fragment of the tibia. 3. Posterior ankle joint. 4. Processus posterior tali. 5. Chisel.

The operation was performed as an outpatient procedure and under general anaesthesia. Initially, the patient was placed in prone position, the foot and ankle were disinfected and a sterile draping was applied. Through a posterolateral portal a 4 mm arthroscope was introduced. The posteromedial portal was used for instrumentation. Inspection of the posterior soft tissues revealed an FHL tendinitis; subsequently FHL release by cutting the flexor retinaculum was performed. The posterior ankle joint showed diffuse synovitis. A posterior joint synovectomy was performed using a 5.5 mm full-radius shaver. The nonunited fragment of the distal posterior edge of the tibia was removed using a combination of a chisel and a 4 mm grasper (Figure 2). Inspection of the articular cartilage of the ankle joint revealed no signs of osteoarthritis. The posterior portals were sutured, and leaving all the instruments on a sterile table, the patient was turned into supine position. The foot and ankle were disinfected again and a fresh sterile draping was applied.

Using standard anteromedial and anterolateral portals an anterior ankle arthroscopy was performed. Again there was extensive synovitis and an additional anterior ankle joint synovectomy was performed. The loose fragments adjacent to the medial malleolus were removed. In addition the anteromedial tibial osteophyte was excised, using a chisel.

A compression bandage was applied and the patient was discharged the same day, starting range of motion exercises from day 0. Weight bearing was allowed after 2 days. On followup 7 days after the surgery, the patient was having an uneventful recovery with limited swelling. Physical rehabilitation program included hydrotherapy from 2 weeks after the procedure. Within 8 weeks he was able to join the soccer league again without restriction. At late follow-up 4.5 years after the operation the patient was without symptoms. On examination, forced plantar flexion did not produce pain posteriorly at the level of the talocrural joint. There were no signs of flexor hallucis longus tendinitis. There was no pain on palpation at the level of the medial malleolus.

## 3. Discussion

Posterior ankle impingement syndrome, os trigonum syndrome, tendinitis of the FHL tendon, posterior tibial tendon, or peroneal tendons, posttraumatic calcifications, bony avulsions, osteochondral defects, ankle and subtalar arthrosis, synovitis, and loose bodies and their combinations, all can be the cause of posterior ankle pain [5]. Arthroscopic evaluation of posterior ankle problems by means of routine ankle arthroscopy using anteromedial, anterolateral, and posterolateral portals is difficult because of the shape of the ankle joint [2]. Only in cases in which the ankle ligaments are very lax, it is possible to visualize and treat posterior ankle pathology by means of anterior portals. Pericapsular and extracapsular posterior ankle pathology cannot be treated by means of routine anterior ankle arthroscopy [5]. Even though in the literature, some investigators have reported technical difficulties in addressing posteromedial lesions and in performing posterior ankle synovectomy [6, 7], we have not experienced this problem, using the 2-portal endoscopic hindfoot technique [4, 8]. In most cases chronic anterior ankle pain is caused by an anterior impingement syndrome, tibiotalar anterior osteophytes, intra-articular foreign bodies, local synovitis, or osteochondral defects [9]. These conditions will markedly reduce ankle function especially limiting dorsiflexion due to an anterior impediment and capsular contracture [9]. Anterior ankle joint pathology can be treated by means of an anterior ankle arthroscopy [10]. In our patient, the patient not only had an anteromedial osteophyte and ossicles, but also joint synovitis and a nonunited fracture of the distal posterior tibial edge combined with a FHL tendinitis. Irritation of the FHL by a nonunited fragment rubbing against it has not been described in the literature before. 

In addressing combined anterior and posterior ankle pathology most surgeons would be reluctant to perform a combined anterior open approach for the anterior pathology and an open posterior approach to solve the posterior problem. This is due to the fact that a combined ventral and dorsal arthrotomy carries a higher risk of complications, and stiffness. An anterior arthroscopic approach with an accessory posterior portal gives good access to the anterior joint, but provides limited access to the posterior joint. A combined anterior arthroscopic and posterior open treatment bears the risk of posterior scar formation, scar tenderness and stiffness [11]. No reports have described the combined posterior and anterior arthroscopic approach in one surgical session, where by the patient is turned from prone to supine position halfway the session. With a well-instructed team in the OR, turning a patient from prone to supine or vice versa, will only take a few minutes whether he is under general or regional anaesthesia. Alternatively, the whole procedure can be performed with the patient in prone position addressing the anterior ankle compartiment with the knee flexed in a 90 degree angle and the ankle in a traction device. Problems with the latter technique are difficult in orientation during arthroscopy and the inability to the dorsiflex the ankle makes this procedure less suitable for combined posterior and anterior ankle arthroscopy. 

We described a patient with combined anterior and posterior ankle pathologies which were treated by means of a combined arthroscopic approach to both the anterior and posterior ankle joint spaces. The obvious advantages are an immediately start of functional rehabilitation and a quick recovery. We have treated several patients since then with similar results.
